# A 19-year-old patient with atypical chronic myeloid leukemia

**DOI:** 10.1007/s00277-020-03992-w

**Published:** 2020-03-19

**Authors:** Philipp Ernst, Björn Engmann, Jochen J. Frietsch, Ulf Schnetzke, Sebastian Scholl, Bernhard Theis, Hans H. Kreipe, Thomas Ernst, Anita Glaser, Torsten Haferlach, Thilo Koch, Andreas Hochhaus, Inken Hilgendorf

**Affiliations:** 1 Abteilung Hämatologie und Internistische Onkologie, Klinik für Innere Medizin II, Universitätsklinikum Jena, Am Klinikum 1, 07740 Jena, Germany; 2 Klinik für Innere Medizin, Burgenlandkreis Klinikum, Naumburg, Germany; 3 Institut für Pathologie, Universitätsklinikum Jena, Jena, Germany; 4 Institut für Pathologie, Medizinische Hochschule Hannover, Hannover, Germany; 5 Institut für Humangenetik, Universitätsklinikum Jena, Jena, Germany; 6 MLL Münchner Leukämielabor, Munich, Germany

Dear Editor,

Atypical chronic myeloid leukemia (aCML) is a rare, aggressive myeloproliferative disorder. The outcome of patients is poor and the therapeutic options are limited. We present the case of a 19-year-old male adolescent who initially had acute abdominal pain due to leukostatic ischemic colitis.

**Fig. 1:**
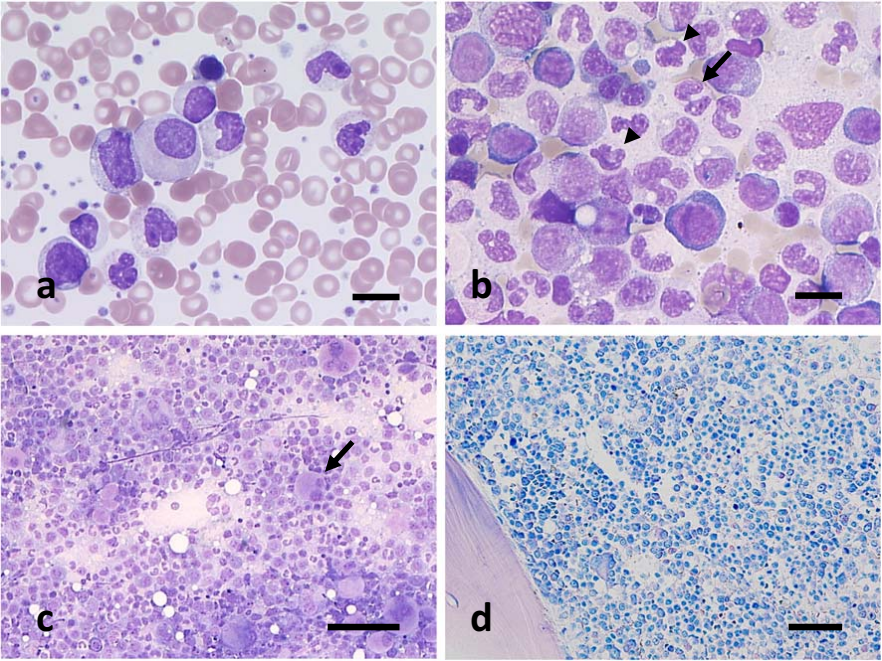
Pappenheim staining of blood smear (**a**) and BM smears (**b**, **c**) and Giemsa staining of pelvic crest trepanation (**d**) at the first presentation in our hospital. The peripheral blood shows leukocytosis with left-shifted maturing granulopoiesis and a marked thrombocytosis (**a**). Dysplastic changes such as pseudo-Pelger forms (arrow in **b**), chromatin clumping (arrowheads in **b**), hypogranulation, and atypical megakaryocytes (arrow in **c**) can be found in the BM. Scale bars = 10 μm (**a**, **b**), 50 μm (**c**, **d**)

A 19-year-old male patient presented to our emergency department with fever, sudden painful abdominal cramps, and bilious vomiting. Computed tomography revealed severe right-sided colitis and a massive hepatosplenomegaly. Furthermore, increased leukocytes 106/nL and platelets 1250/nL as well as a mild anemia were detected. The differential blood count showed 41% segment pithy granulocytes, 15% rod pithy granulocytes, 5% metamyelocytes, 23% myelocytes, 2% promyelocytes, 4% blasts, 1% basophilic granulocytes, 1% eosinophilic granulocytes, and 4% erythroblasts (Fig. 1a). Cytological examination of the bone marrow (BM) showed hypercellularity with an increased and dysplastic granulopoiesis and megakaryopoiesis as well as a relatively diminished and also dysplastic erythropoiesis (Fig. 1b, c). The proportion of non-erythroid blasts was 10%. Histological findings showed an almost complete disappearance of adipose tissue due to myeloproliferation. The increased granulopoiesis still preserved maturation without involvement of the eosinophil series. Signs of dysplasia in megakaryopoiesis were detectable without finding micromegakaryocytes (Fig. 1d). Cytogenetic analysis by fluorescence in situ hybridization (FISH) failed to detect *BCR-ABL1* gene fusion or *FGFR1* rearrangements but did find a trisomy 8 (47,XY,+8). Moleculargenetic analysis excluded mutations for *BCR-ABL1*, *JAK2*, *CALR*, *MPL*, *ASXL1*, *CBL*, *CSFR3*, *ETNK1*, *EZH2*, *SETBP1*, and *TET2*. However, mutations were found in the genes *SF3B1* and *NF1*.

Based on the evidence of an as yet unidentified myeloproliferative disease and the suspected colitis associated with it, oral cytoreductive therapy was started at 1 g hydroxyurea daily. In terms of differential diagnosis, it was suspected that the patient was suffering from a *BCR-ABL1* negative myeloproliferative/myelodysplastic disease. In contrast to *BCR-ABL1* positive CML, the absence of *BCR-ABL1* transcript as well as multilinear dysplasia with prominent dysgranulopoiesis and the absence of basophilia were conspicuous. Since leukocytosis with more than 10% granulocytic precursors, hypercellular BM, and less than 20% blasts in peripheral blood and BM were also detectable, the patient met the criteria for aCML according to the 2016 WHO classification.

**Fig. 2:**
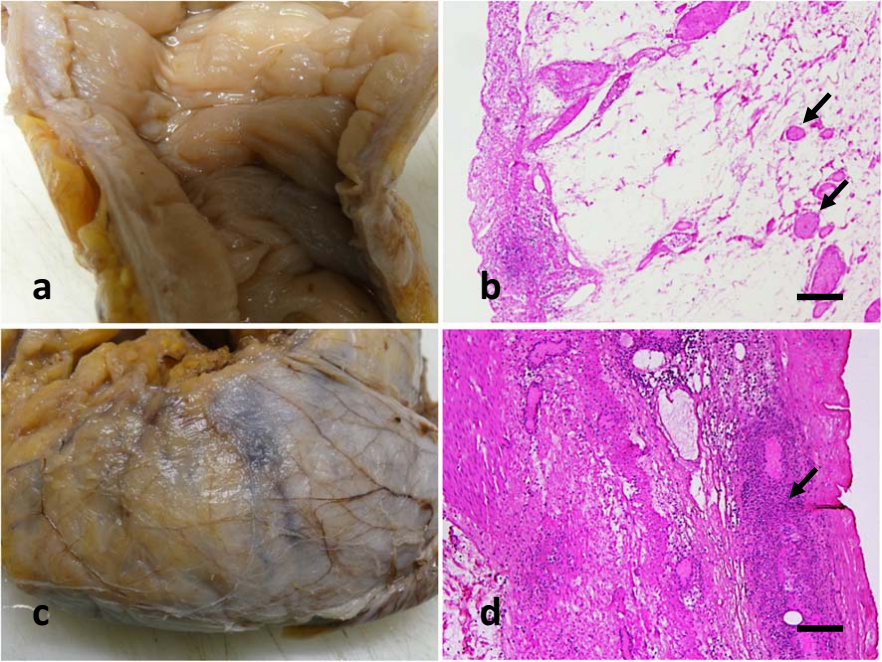
Preparation after right hemicolectomy (**a**, **c**) and HE staining of histological sections of the colonic wall (mucosa left in **b**, serosa right in **d**). The mucosa shows a strong edematous wall thickening (**a**) as well as a complete necrosis with inflammatory infiltrates, strongly edematous and widened submucosa and capillary thrombi (arrows in **b**). In the serosa, there is an inflammation-related fibrosis (**c**) and the entire intestinal wall is interspersed with a florid inflammatory infiltrate (here predominantly neutrophilic granulocytes in the area of subserosa and serosa) (arrow in **d**). Scale bars = 200 μm

Two days after hospitalization, he developed a septic condition with clinical suspicion of an acute abdomen. Due to a diffuse peritonitis and a suspected perforation of the right hemicolon, an emergency hemicolectomy on the right was performed. Severe edematous bowel wall with ulcerous mucosal defects and inflammatory infiltrates of the mucosa (Fig. 2a, b), thrombosis of the submucosal capillaries, and fibrinous-purulent inflammation of the serosa corresponding to ischemic colitis with peritonitis appeared histologically (Fig. 2c, d).

**Fig. 3:**
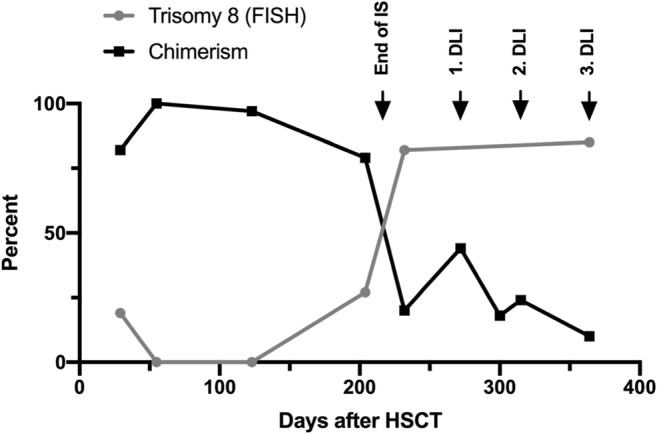
History of chimerism (percentage of donor cells) and FISH detectable cells with trisomy 8 (percentage of all BM cells) after hematopoietic blood stem cell transplantation (HSCT). After initial reduction of immunosuppression (IS) on day + 29, a chimerism of 100% could be achieved. After another drop in chimerism and increase in trisomy 8 content from day + 204, IS was rapidly stopped on day + 217. Due to the inadequate graft-versus-leukemia (GvL) effect, the patient received donor lymphocytes infusions (DLI) on day + 272, + 315, and + 364 (5 × 10^6^, 1 × 10^7^, and 1.7 × 10^7^ CD3-positive cells/kg bwt, respectively) with only moderate success

In addition to hydroxyurea, cytoreductive therapy with cytarabine 100 mg/m^2^ daily was supplemented for 3 days at 4-week intervals for 3 months resulting in partial remission. Five months after diagnosis, the patient received an allogeneic hematopoietic blood stem cell transplantation (HSCT) from a 9/10 HLA-mismatched unrelated donor. Due to poor general condition with pulmonary aspergillosis, gluteal infection, immobility, and renal impairment, he received a reduced toxicity conditioning with cytarabine, treosulfan, and fludarabine. On day + 29, the BM showed a regenerating hematopoiesis and a chimerism of 82%. Furthermore, in about 20–35% of interphases, the clone specific trisomy 8 could be detected by FISH. Therefore, the immunosuppressive therapy was rapidly reduced so that no trisomy 8 and a donor proportion of 100% could be achieved on day + 55 (Fig. 3).

However, 6 months after HSCT, a cytogenetic recurrence and 1 month later, a hematological recurrence were detected, despite discontinuation of immunosuppression. Therapy with hydroxyurea was started again. Subsequently, three donor lymphocyte infusions (DLI, 5 × 10^6^, 1 × 10^7^, and 1.7 × 10^7^ CD3-positive cells/kg bwt, respectively) were performed to enhance the graft-versus-leukemia (GvL) effect. Despite relapse, the general condition improved and the patient had fully regenerated renal function. Therefore, a second allogeneic HSCT was planned. However, on day + 409, he presented again with progressive pains due to displacing growth of his spleen. Intravenous cytarabine (100 mg/m^2^ for 3 days) was administered as differential leukocyte count revealed an excessive increase in immature cells. Unfortunately, the patient deceased within 3 days (on day + 416) on relapse with septic shock, disseminated intravascular coagulation, and multiorgan failure.

Due to the aggressive biology of the disease as in this case, the aim is to HLA-typify aCML patients for allogeneic HSCT as early as possible. If they are suitable for HSCT and if no cytoreduction is initially necessary, it is possible to proceed directly to HSCT if a donor is available. However, in most cases, cytoreductive therapy is required. Therefore, hypomethylating therapy as well as pegylated interferon α or hydroxyurea is recommended prior to transplantation when the aCML clones have an inconspicuous mutation status [1]. If potentially actionable, myeloid mutations are detectable (*JAK2*, *CSF3R*, *RAS*); therapy with a targeted agent is an option. In contrast to *BCR-ABL1* positive CML, however, no selective targeted therapy with tyrosine kinase inhibitors is available. Due to the patient’s hyperleukocytosis with associated ischemic colitis, cytoreductive therapy was initially necessary, and because the worsened overall condition, including the surgical formation of a terminal ileostomy and pulmonary and soft tissue infections, no timely HSCT was possible. The time from diagnosis to HSCT was 6 months; the EBMT risk score was 2 points (HLA-compatible unrelated donor, accelerated phase before transplantation) and the HCT-CI 4 points (mild hepatic and pulmonary comorbidity, active infection).

The median overall survival (OS) of aCML patients is between 12 and 25 months [2, 3]. In 37%, aCML transforms into secondary acute myeloid leukemia (sAML), with a median time to transformation of 11.2 months. Unfavorable prognostic factors for the OS are an initial increased white blood cell count (> 40/nL), increased immature precursors in peripheral blood, female gender, and high patient age [3]. In a retrospective analysis of aCML patients after HSCT, 87% of the patients had a complete remission, 6.5% had a partial response, and 6.5% were non-responders. After 5 years, relapse-free survival (RFS) was 36% and mean OS 51%. In the subgroup analysis, a young recipient age (< 45 years) and a low EBMT risk score (1–2 points) as well as stem cells from an unrelated HLA-compatible donor had a favorable effect on the 5-year OS [4]. Despite the low EBMT risk score, the patient achieved only a partial response on day + 29 after transplantation. Certainly, the loss of time until HSCT due to severe infections and the need to reduce the toxicity of the conditioning regimen due to comorbidities and reduced general health condition were disadvantages. However, studies found no significant advantage in early HSCT within 6 months compared with transplantation between 6 and 12 months or more than 12 months after diagnosis [4].

One or two chromosomal aberrations (e.g., trisomy 8, del (20q), -7 / 7q or isochromosomes 17q) are found in 80% of patients [5]. Moleculargenetic changes are nonspecific. The most commonly affected genes (> 20%) are *SETBP1*, *ASXL1*, *N/K-RAS*, *SRSF2*, and *TET2* and less frequently (< 10%) *CBL*, *CSFR3*, *JAK2*, and *ETNK1* [1]. The mutation in *Splicing factor 3B subunit 1* (*SF3B1*) gene, which was found in the patient, is most highly prevalent in MDS patients with ring sideroblasts (MDS-RS-SLD or MDS-RS-MLD) and is usually associated with a favorable prognosis [6]. Constitutional mutations in *neurofibromin 1* (*NF1*) gene, which occurs in the RAS pathway, predispose patients to malignant myeloid disorders such as juvenile myelomonocytic leukemia (JMML), chronic myelomonocytic leukemia (CMML), and acute myeloid leukemia (AML), regardless of whether clinical signs of neurofibromatosis exist [7]. JMML is a pediatric myelodysplastic syndrome in which subclonal *NF1* mutations are detectable in fewer than 5% of cases [8]. In AML patients, mutations in *NF1* gene are suggestive of an adverse prognostic impact [9]. Whether these two additional mutations in the *SF3B1* and *NF1* genes, as found in the patient, are driver mutations that adversely affect the aCML is conceivable but is currently still unclear.
